# Supplementary material for: Association Between Dietary Intake, Meal Patterns, and Malnutrition Risk Among Community-Dwelling Elderly in Northern Thailand: A Cross-Sectional Study
Source: Nutrients. 2025 Nov 12;17(22):3537. doi: 10.3390/nu17223537 (PMC12655047; doi:10.3390/nu17223537)
Supplement: Supplementary file 1 [file nutrients-17-03537-s001.zip › nutrients-3912982-supplementary.pdf]

Table S1. Descriptive characteristics of the participants according to nutritional status

Characteristics	Total (N=200)	Malnutrition risk (N=46)	Well-nourished (N=154)	*p*-value
Age (years), mean ± SD	68.5 ± 6.5	73.2 ± 8.5	67.1 ± 5.0	**<0.001**[a]
60-69 years	127 (63.5)	18 (39.1)	109 (70.8)	**<0.001**[c]
70-79 years	61 (30.5)	18 (39.1)	43 (27.9)	
≥80 years	12 (6.0)	10 (21.7)	2 (1.3)	
Sex: Female	122 (61.0)	26 (56.5)	96 (62.3)	0.478[b]
Single/Separated/Divorced/Widowed	72 (36.0)	22 (37.8)	50 (32.5)	0.057[b]
Education level ≤ Elementary school	159 (79.5)	41 (89.1)	118 (76.6)	0.065[b]
Unemployment	109 (54.5)	33 (71.7)	76 (49.4)	**0.007**[b]
Income ≤ 5,000 bath/month	116 (58.0)	40 (87.)	76 (49.4)	**<0.001**[b]
Utilized Universal Coverage scheme (UC)	145 (72.5)	40 (86.96)	105 (68.18)	**0.012**[b]
Multimorbidity	77 (38.5)	18 (39.1)	59 (38.3)	0.920[b]
Polypharmacy (≥5 types of medication used)	13 (6.5)	5 (10.9)	8 (5.2)	0.181[c]
Psychiatric disorders*	3 (1.5)	3 (6.5)	0 (0)	**0.012**[c]
Cognitive impairment	24 (12.00)	17 (36.96)	7 (4.55)	**<0.001**[b]
Active smokers	7 (3.5)	3 (6.5)	4 (2.6)	0.200[c]
Alcohol drinkers	27 (13.5)	5 (13.0)	18 (13.6)	0.918[b]
Physical activity, MET-minutes, median (IQR)	790 (350, 1400)	360 (0, 960)	800 (440, 1600)	**<0.001**[d]
TEE (kcal), median (IQR)	1744.7 (1429.3, 2068.5)	1379.8 (1171.1, 1750.6)	1840.5 (1515.9, 2135.6)	**<0.001**[d]
Sleep duration (hours), median (IQR)	9 (9, 10)	9.75 (9, 10.5)	9 (0, 10)	0.104[d]
SPPB score, median (IQR)	11 (8, 12)	7 (3, 10)	11 (10, 12)	**<0.001**[d]
10-12 (well physical performance)	140 (70.0)	16 (34.8)	124 (80.5)	**<0.001**[b]
0-9 (poor physical performance)	60 (30.0)	30 (65.2)	30 (19.5)	
Anthropometrics, mean ± SD				
Weight (kg)	55.1 ± 11.7	46 ± 8.5	57.9 ± 11.1	**<0.001**[a]
Height (cm)	155.8 ± 8.3	154.1 ± 7.3	156.3 ± 8.5	0.1161[a]
BMI (kg/m^2)	22.61 ± 3.74	19.37 ± 3.19	23.58 ± 3.33	**<0.001**[a]
MUAC (cm)	25.9 ± 5.4	20.7 ± 3.8	27.5 ± 4.7	**<0.001**[a]
WC (cm)	79.1 ± 10.4	72.2 ± 8.8	81.2 ± 9.9	**<0.001**[a]
CC (cm)	32.4 ± 4.5	29.0 ± 3.3	33.4 ± 4.2	**<0.001**[a]

SD, standard deviation; IQR, interquartile range; TEE, total energy expenditure; SPPB, short physical performance battery; BMI, body mass index; MUAC, mid upper arm circumference; WC, waist circumference; CC, calf circumference

Significant results are shown in bold.

[a] Independent t-test, [b] Chi-square test, [c] Fisher's exact test, [d] Mann-Whitney U test

* Psychiatric disorders including depression, panic disorder, alcohol dependent

Table S2. Association between Dietary intakes and Meal pattern with Nutritional status

Characteristics	Total (N=200)	Malnutrition risk (N=46)	Well-nourished (N=154)	*p*-value
Daily calories intake (kcal), mean ± SD	1609.77 ± 505.43	1453.27 ± 546.98	1656.51 ± 484.47	**0.016[a]**
Breakfast calories intake	487.67 ± 309.63	486.14 ± 363.43	488.13 ± 292.99	0.970[a]
Lunch calories intake	513.62 ± 234.04	465.68 ± 231.61	527.93 ± 233.60	0.114[a]
Dinner calories intake	579.20 ± 316.461	491.15 ± 292.43	605.50 ± 319.50	**0.031[a]**
Protein daily intake (gm), median (IQR)	91.03 (63.61, 124.31)	100.84 (51.37, 127.35)	87.81 (64.12, 123.80)	0.972[d]
Breakfast	22.69 (10.12, 46.16)	21.46 (7.55, 53.16)	23.03 (10.48, 42.66)	0.712[d]
Lunch	25.26 (13.66, 42.84)	22.58 (11.09, 43.03)	25.91 (14.30, 42.66)	0.544[d]
Dinner	31.63 (17.31, 53.15)	31.89 (12.80, 46.76)	31.63 (18.48, 53.64)	0.387[d]
Carbohydrate daily intake (gm), median (IQR)	148.13 (109.56, 198.36)	116.79 (85.41, 165.98)	154.30 (122.43, 202.73)	**0.002[d]**
Breakfast	46.94 (23.89, 65.01)	35.93 (15.03, 63.77)	49.28 (27.21, 65.40)	**0.030[d]**
Lunch	50.97 (30.02, 76.32)	45.08 (23.41, 68.45)	52.36 (35.76, 76.65)	0.075[d]
Dinner	47.08 (32.06, 66.87)	39.01 (24.06, 55.04)	50.97 (35.16, 69.65)	**0.006[d]**
Fat daily intake (gm), median (IQR)	54.44 (36.59, 83.52)	51.21 (31.26, 80.99)	54.58 (38.78, 84.36)	0.344[d]
Breakfast	10.75 (3.00, 30.46)	10.58 (3.05, 30.27)	10.85 (2.95, 30.55)	0.977[d]
Lunch	13.17 (5.88, 28.37)	11.86 (3.81, 27)	13.17 (6.74, 29.77)	0.209[d]
Dinner	19.17 (7.44, 34.50)	17.15 (2.77, 31.13)	19.31 (8.42, 34.63)	0.164[d]
Overnight fasting (hrs), median (range)	13.5 (10.5-23)	14 (11.5-23)	13.5 (10.5-17.5)	**<0.001[d]**
Eating episodes* (times), median (range)	3 (1-6)	3 (1-4)	3 (2-6)	**<0.001[d]**
Meal skipper**, N (%)	34 (17.00)	14 (30.43)	20 (12.99)	**0.006[b]**
Breakfast skipper	25 (12.50)	9 (19.57)	16 (10.39)	0.099[b]
Lunch skipper	4 (2.00)	2 (4.35)	2 (1.30)	0.2270[c]
Dinner skipper	6 (3.00)	4 (8.70)	2 (1.30)	**0.026[c]**

* An 'eating episode' is considered if a minimum of 50 calories are consumed, and the meal interval is more than 1 hour (count both meal and snack)

** Meal skipper is classified if consuming less than 200 calories within designated timeframes: 6:00 AM – 9:00 AM (breakfast), 11:00 AM – 2:00 PM (lunch), or 5:00 PM – 8:00 PM (dinner).

Significant results are shown in bold.

[a] Independent t-test, [b] Chi-square test, [c] Fisher's exact test, [d] Mann-Whitney U test

Table S3. Prevalence of inadequate nutrients intake according to the Thai Dietary Reference Intake 2020

Characteristics	Total (N=200)	Malnutrition risk (N=46)	Well-nourished (N=154)	*p*-value
**Macronutrients**				
TEI (kcal/d), mean ± SD	1609.8 ± 505.4	1453.3 ± 547.0	1656.5 ± 484.5	**0.016[a]**
TEI ≥ TEE	77 (38.5)	23 (50)	54 (35.1)	0.068[b]
TEI < TEE	123 (61.5)	23 (50)	100 (64.9)	
Protein (g/d), mean ± SD	99.3 ± 46.9	98.2 ± 50.2	99.6 ± 46.0	0.855[a]
Percentage of TEI	23.34 (19.76, 28.70)	24.79 (20.35, 29.15)	22.97 (19.75, 28.44)	0.158[d]
≥10% of TEI	199 (99.5)	46 (100)	153 (99.4)	1.000[c]
<10% of TEI	1 (0.5)	0 (0)	1 (0.7)	
Carbohydrates (g/d), mean ± SD	159.5 ± 72.8	132.4 ± 67.2	167.6 ± 72.6	**0.004[a]**
Percentage of TEI	40.22 (29.22, 52.99)	35.72 (26.63, 52.84)	40.62 (29.80, 53.13)	0.170[d]
≥45% of TEI	78 (39.0)	14 (30.4)	64 (41.6)	0.175[b]
<45% of TEI	122 (61.0)	32 (69.6)	90 (58.4)	
Fat (g/d), mean ± SD	62.8 ± 36.7	59.0 ± 39.0	63.9 ± 36.1	0.427[a]
Percentage of TEI	33.15 (23.41, 44.47)	33.53 (24.09, 46.82)	33.06 (23.06, 42.93)	0.778[d]
≥20% of TEI	168 (84.0)	39 (84.8)	129 (83.8)	0.869[b]
<20% of TEI	32 (16.0)	7 (15.2)	25 (16.2)	
**Micronutrients**				
Vitamin A (mcg/d), median (range)	218.19 (78.21, 459.75)	218.83 (75.63, 423.71)	218.19 (83.05, 475.24)	0.678[d]
< RDA	178 (89.0)	42 (91.3)	128 (83.1)	0.172[b]
Vitamin C (mg/d), median (range)	42.14 (16.26, 92.24)	36.20 (18.91, 88.74)	46.83 (15.68, 102.36)	0.477[d]
< RDA	147 (73.5)	35 (76.1)	112 (72.7)	0.651[b]
Vitamin E (mg/d), median (range)	0.91 (0.09, 2.44)	0.12 (0, 1.20)	1.05 (0.16, 2.62)	**<0.001[d]**
< RDA	197 (98.5)	46 (100)	151 (98.1)	1.000[c]
Thiamine (mg/d), median (range)	1.37 (0.76, 2.37)	1.21 (0.53, 2.16)	1.40 (0.83, 2.46)	0.144[d]
< RDA	82 (41.0)	22 (45.8)	60 (39.0)	0.283[b]
Riboflavin (mg/d), median (range)	1.26 (0.95, 1.89)	1.29 (0.87, 2.21)	1.24 (0.98, 1.86)	0.949[d]
< RDA	83 (41.5)	20 (43.5)	63 (40.9)	0.756[b]
Niacin (mg/d), median (range)	15.49 (11.24, 23.66)	15.36 (11.69, 23.32)	15.49 (11.22, 23.68)	0.951[d]
< RDA	95 (47.5)	21 (45.7)	74 (48.1)	0.775[b]
Vitamin B6 (mg/d), median (range)	0.22 (0.04, 0.39)	0.15 (0, 0.33)	0.22 (0.06, 0,40)	0.141[d]
< RDA	194 (97.0)	46 (100)	148 (96.1)	0.340[c]
Vitamin B12 (mcg/d), median (range)	0.01 (0, 0.92)	0.01 (0, 1.02)	0.01 (0, 0.92)	0.957[d]
< RDA	179 (89.5)	41 (89.1)	138 (89.6)	1.000[c]
Calcium (mg/d), median (range)	313.34 (179.48, 582.14)	292.33 (136.50, 603.50)	318.53 (213.79, 570.76)	0.357[d]
< RDA	185 (92.5)	44 (95.7)	141 (91.6)	0.528[c]
Phosphate (mg/d), median (range)	728.40 (521.91, 1052.37)	801.60 (529.85, 1134.29)	726.29 (517.74, 1004.05)	0.601[d]
< RDA	96 (48.0)	21 (45.7)	75 (48.7)	0.716[b]
Magnesium (mg/d), median (range)	**19.99 (5.76, 58.57)**	**10.68 (0.53, 39.51)**	**24.55 (7.56, 63.79)**	**0.008[d]**
< RDA	199 (99.5)	46 (100)	153 (99.4)	1.000[c]
Iron (mg/d), median (range)	11.29 (8.00, 14.33)	9.67 (6.98, 15.14)	11.69 (8.24, 15.52)	0.061[d]
< RDA	90 (45.0)	27 (58.7)	63 (40.9)	**0.033[b]**
Zinc (mg/d), median (range)	**5.99 (4.08, 8.18)**	**4.57 (3.28, 6.95)**	**6.39 (4.31, 8.40)**	**0.010[d]**

< RDA	168 (84.0)	41 (89.1)	127 (82.5)	0.279[b]
Selenium (mcg/d), median (range)	14.17 (0.31, 35.32)	9.63 (0, 31.44)	16.05 (0.31, 36.12)	0.087[d]
< RDA	172 (86.0)	41 (89.1)	131 (85.1)	0.486[b]
Copper (mg/d), median (range)	0.68 (0.49, 0.86)	0.65 (0.44, 0.82)	0.68 (0.53, 0.91)	0.194[d]
< AI	185 (92.5)	42 (91.3)	143 (92.9)	0.752[c]
Sodium (mg/d), median (range)	3744.61 (2524.8, 5898.36)	3193.48 (2004.67, 5102.23)	3979.27 (2738.48, 5933.14)	0.039[d]
< RDA	1 (0.5)	0 (0)	1 (0.7)	1.000[c]
> UL	185 (92.5)	43 (93.5)	142 (92.2)	0.774[b]
Potassium (mg/d), median (range)	1455.11 (955.27, 2108.96)	1469.29 (822.48, 2134.09)	1455.11 (992.08, 2108.6)	0.599[d]
< RDA	147 (73.5)	33 (71.7)	114 (74.0)	0.758[b]
Dietary fiber (g/d), median (range)	**9.05 (4.63, 13.29)**	**8.81(3.09, 11.45)**	**9.39 (5.50, 13.69)**	**0.034[d]**
< RDA	188 (94.0)	45 (97.8)	143 (92.9)	0.303[c]

RDA, recommended dietary allowances; AI, adequate intakes; TEI, total energy intakes, TEE, total energy expenditure

Significant results are shown in bold.

[a] Independent t-test, [b] Chi-square test, [c] Fisher’s exact test, [d] Mann-Whitney U test

Table S4. Association of dietary intake (divided by the standard deviation) and meal pattern with malnutrition risk

Variables	Model 0		Model 1		Model 2		Model 3	
	OR (95% CI)	*p*-value	OR (95% CI)	*p*-value	OR (95% CI)	*p*-value	OR (95% CI)	*p*-value
TEI (SD=505.43 kcal/d)	0.66 (0.47-0.93)	**0.018**	0.70 (0.47-1.04)	0.081	0.81 (0.52-1.25)	0.336	-	-
**Macronutrients**								
Protein (SD=46.88 g/d)	0.96 (0.70-1.35)	0.854	0.98 (0.66-1.45)	0.917	1.16 (0.75-1.77)	0.508	1.77 (0.97-3.23)	0.061
Carbohydrate (SD=72.78 g/d)	0.56 (0.37-0.84)	**0.005**	0.61 (0.38-0.98)	**0.039**	0.76 (0.46-1.23)	0.261	0.80 (0.47-1.36)	0.416
Fat (SD=36.72 g/d)	0.87 (0.62-1.22)	0.425	0.87 (0.58-1.31)	0.509	0.86 (0.56-1.32)	0.489	1.03 (0.52-2.03)	0.932
**Micronutrients**								
Vitamin A (SD=4294.99 mcg/d)	1.01 (0.73-1.39)	0.946	0.99 (0.72-1.37)	0.965	1.04 (0.73-1.47)	0.839	1.05 (0.75-1.47)	0.763
Vitamin C (SD=142.53 mg/d)	0.77 (0.45-1.32)	0.343	0.65 (0.31-1.36)	0.252	0.69 (0.31-1.52)	0.357	0.73 (0.33-1.62)	0.427
Vitamin E (SD=5.82 mg/d)	0.24 (0.06-0.96)	**0.044**	0.07 (0.01-0.41)	**0.003**	0.05 (0.01-0.32)	**0.001**	0.05 (0.01-0.33)	**0.002**
Thiamine (SD=2.89 mg/d)	0.60 (0.27-1.32)	0.203	0.81 (0.43-1.52)	0.514	0.85 (0.45-1.59)	0.608	0.91 (0.51-1.63)	0.758
Riboflavin (SD=0.99 mg/d)	1.03 (0.74-1.42)	0.873	0.97 (0.69-1.36)	0.833	1.03 (0.71-1.50)	0.858	1.18 (0.78-1.77)	0.435
Niacin (SD=9.50 mg/d)	0.97 (0.69-1.35)	0.840	1.06 (0.73-1.54)	0.747	1.25 (0.82-1.89)	0.303	1.58 (0.96-2.60)	0.072
Vitamin B6 (SD=0.42 mg/d)	0.64 (0.39-1.07)	0.086	0.55 (0.30-1.00)	0.050	0.52 (0.27-1.02)	0.056	0.54 (0.28-1.05)	0.070
Vitamin B12 (SD=4.84 mcg/d)	1.17 (0.88-1.56)	0.272	1.11 (0.80-1.52)	0.537	1.13 (0.78-1.63)	0.512	1.17 (0.82-1.67)	0.395
Calcium (SD=926.57 mg/d)	0.77 (0.38-1.56)	0.472	0.46 (0.16-1.31)	0.146	0.43 (0.14-1.31)	0.138	0.47 (0.15-1.49)	0.200
Phosphate (SD=440.92 mg/d))	1.13 (0.82-1.56)	0.447	1.06 (0.76-1.57)	0.639	1.14 (0.77-1.69)	0.511	1.35 (0.86-2.13)	0.192
Magnesium (SD=53.02 mg/d)	0.74 (0.48-1.13)	0.163	0.56 (0.34-0.92)	**0.022**	0.68 (0.41-1.13)	0.134	0.69 (0.41-1.16)	0.167
Iron (SD=8.84 mg/d)	0.58 (0.35-0.97)	**0.038**	0.62 (0.36-1.09)	0.096	0.68 (0.40-1.15)	0.151	0.71 (0.40-1.25)	0.233
Zinc (SD=4.37 mg/d)	0.65 (0.42-1.03)	0.064	0.81 (0.52-1.26)	0.340	0.92 (0.58-1.46)	0.716	1.00 (0.62-1.63)	0.990
Selenium (SD=32.58 mcg/d)	0.71 (0.46-1.10)	0.129	0.65 (0.39-1.07)	0.088	0.62 (0.36-1.07)	0.084	0.64 (0.37-1.11)	0.110
Copper (SD=1.17 mg/d)	1.05 (0.77-1.42)	0.774	1.03 (0.75-1.40)	0.877	1.08 (0.77-1.52)	0.646	1.12 (0.81-1.56)	0.486
Sodium (SD=3599.05 mg/d)	0.61 (0.38-0.98)	**0.042**	0.66 (0.38-1.15)	0.139	0.56 (0.31-1.04)	0.067	0.58 (0.31-1.11)	0.101
Potassium (SD=962.65 mg/d)	0.94 (0.67-1.31)	0.709	0.86 (0.58-1.27)	0.449	0.90 (0.60-1.37)	0.632	0.97 (0.62-1.53)	0.909
Dietary fiber (SD=8.24 g/d)	0.72 (0.48-1.07)	0.105	0.65 (0.41-1.05)	0.077	0.65 (0.40-1.07)	0.089	0.66 (0.40-1.09)	0.108
**Meal pattern**								
Overnight fasting duration	1.86 (1.35-2.54)	**<0.001**	2.04 (1.41-2.96)	**<0.001**	2.16 (1.43-3.26)	**<0.001**	2.14 (1.42-3.25)	**<0.001**
Meal skipper	2.99 (1.41-6.35)	**0.004**	2.58 (1.02-6.54)	**0.046**	2.11 (0.80-5.58)	0.133	1.93 (0.69-5.43)	0.213
Eating episodes	0.20 (0.08-4.80)	**<0.001**	0.15 (0.05-0.43)	**0.001**	0.16 (0.05-0.49)	**0.001**	0.16 (0.05-0.50)	**0.002**

TEI, total energy intake

Significant results are shown in bold.

Model 0: Crude

Model 1: adjusted for age, sex, marital status, low income, smoking, alcohol use

Model 2: adjusted for variables in model 2 plus cognitive impairment, poor physical performance

Model 3: adjusted for variables in model 3 plus total energy intake.
